# Relationship between impaired work function and coping behaviors in workers with low back pain

**DOI:** 10.1002/1348-9585.12272

**Published:** 2021-08-30

**Authors:** Kosuke Sakai, Tomohisa Nagata, Masako Nagata, Shigeyuki Kajiki, Yoshihisa Fujino, Koji Mori

**Affiliations:** ^1^ Department of Occupational Health Practice and Management Institute of Industrial Ecological Sciences University of Occupational and Environmental Health Kitakyushu Japan; ^2^ Department of Environmental Epidemiology Institute of Industrial Ecological Sciences University of Occupational and Environmental Health Kitakyushu Japan

**Keywords:** behavior, low back pain, presenteeism, work function impairment

## Abstract

**Objectives:**

The aim of the present study was to clarify the relationship between work functional impairment levels and three coping behaviors of workers with low back pain, which were about seeking medical attention, taking over‐the‐counter drugs, and taking self‐care.

**Methods:**

We conducted a cross‐sectional study on 14 Japanese companies in 2016. Work function impairment was measured using the Work Functioning Impairment Scale. Logistic regression analyses were conducted for the three coping behaviors and odds ratios (ORs) calculated for work functional impairment levels.

**Results:**

We analyzed 2232 subjects; 226 were women and 790 worked on production lines. 688 workers had sought medical attention, 436 had taken over‐the‐counter medication, and 1225 had engaged in self‐care. Those seeking medical attention were associated with severe work function impairment compared with no work function impairment (adjusted OR = 2.84, 95% confidence interval: 1.82–4.45, *p* < .001). We observed a trend for the association between over‐the‐counter drug use with high levels of work function impairment (adjusted OR: 1.19 for low, 1.35 for moderate, 1.65 for severe). There was no apparent relationship between self‐care and the degree of work functional impairment.

**Conclusion:**

In workers with low back pain, severe work functional impairment may promote medical attention and over‐the‐counter medication use, but it would not encourage self‐care, such as stretching or exercise. Therefore, workplaces need to provide special support to help them take care of themselves. Therefore, it is desirable to provide good support for self‐care in the workplace.

## 1 INTRODUCTION

The higher average age of workers in aging societies means that more people in the workplace have health problems. A comprehensive study assessing global prevalence, incidence, and years lived with disability for health problems identified low back pain as the biggest health challenge.^1^ As the global population ages, the number of people with chronic diseases, and the social burden of back pain, will increase.^2^

The effect of health problems on work functioning is defined as work function impairment, and loss of productivity caused by work function impairment is defined as presenteeism.^3^ Productivity loss caused by presenteeism differs according to the type of health disorder. A study in Japan showed that musculoskeletal disorders and psychiatric disorders lead to greater presenteeism^4^; 60% of respondents with musculoskeletal pain experienced back pain.^4^ If musculoskeletal pain becomes chronic, it can lead to an impaired work function.^5^ The most common type of musculoskeletal pain associated with work function impairment is low back pain.^6^ A combination of pain‐induced depressive symptoms and pain‐induced work difficulty synergistically causes substantial productivity loss from presenteeism.^7^

Work function impairment is a serious issue, and many types of workplace health promotion have been conducted to prevent and improve work function impairment and reduce the cost of presenteeism.^8^ Although some intervention studies in the workplace aimed at improving occupational dysfunction due to low back pain have been reported, their effectiveness is limited.^9^, ^10^

Before discussing workplace interventions to improve work dysfunction for workers with low back pain, it is important to review the coping behaviors that workers with low back pain may engage in. Individuals with back pain should seek medical attention at a relatively early stage. If there are no red flags, such as vertebral fractures or suspicion of cancer, physical therapy, and self‐management are often recommended.^11^ Patients with acute low back pain tend to rest because of the pain caused by physical movement, but it has been found that continuing activities of daily living, even in the acute stage, can accelerate return to work.^12^ Therefore, education is needed to help health care providers to correct misconceptions and encourage appropriate self‐care. A previous study found that patients with low back pain who visited a medical institution during the acute or subacute phase and received education about low back pain experienced lower fear and anxiety.^13^

Some individuals use over‐the‐counter medications as a coping behavior. Non‐steroidal anti‐inflammatory drugs and acetaminophen are recommended for symptomatic relief of low back pain and can be purchased over the counter.^14^ Although there are benefits and disadvantages to long‐term use of over‐the‐counter medications, their temporary use is recommended for self‐management because of its effectiveness.^15^

The most important coping behavior is to prevent the recurrence and chronicity of low back pain through self‐coping. Strength training and stretching to improve muscle strength and flexibility in the lower back are effective in preventing recurrence and chronicity of low back pain.^16^ Various self‐coping methods, such as yoga, tai chi, and Pilates, have been recommended^17^, ^18^ Massage is also recommended in the acute phase owing to its minimal adverse effects, although the evidence for its effectiveness is weak.^19^

As mentioned above, people with back pain should take the following actions: visit a medical institution, use over‐the‐counter medications, or engage in self‐care. Companies may need to provide support to workers with low back pain to help them to select appropriate coping behaviors to improve their work function. However, there is a lack of research on work dysfunction and coping behaviors as a background to consider in developing effective support strategies. Therefore, the aim of the present study was to clarify the relationship between work functional impairment levels and three coping behaviors of workers with low back pain.

## 2 METHODS

### 2.1 Study design

This was a cross‐sectional study of workers in 14 large companies (13 manufacturing companies and one medical and welfare service company) in Japan. We analyzed data from a self‐administered questionnaire survey conducted in 2016. The questionnaire was administered both on paper and online. The response period was from July to September 2016.

### 2.2 Selection of subjects for analysis

To identify subjects with low back pain, respondents were asked about the presence of the following health problems and disorders during the past month: allergic diseases, skin diseases, and itching, disorders caused by infectious diseases, gastrointestinal disorders, pain and discomfort in limbs and joints, low back pain, neck disorders and stiff shoulders, headache, dental disorders, mental disorders, sleep disorders, general malaise, eye problems, and other problems.^20^ Respondents were asked to select all symptoms that applied to them. In the following question, respondents who selected multiple symptoms were asked to select the one health problem that had the greatest effect on their work. The subjects of this study were those who selected low back pain as the symptom that most affected their work.

### 2.3 Assessment of work function impairment level

The questionnaire also included the Work Functioning Impairment Scale (WFun).^3^ This scale evaluates workers’ health‐related disability during work time and contains seven items: (1) I have not been able to behave socially; (2) I have not been able to maintain the quality of my work; (3) I have had trouble thinking clearly; (4) I have taken more rests during my work; (5) I have felt that my work is not going well; (6) I have not been able to make rational decisions; and (7) I have not been proactive about my work. For each item, respondents were asked to select the most appropriate response from the following five options: almost every day (5 points), two or more days a week (4 points), about one day a week (3 points), one or more days a month (2 points), and not at all (1 point). The total possible score ranges from 7 to 35. Higher scores indicate greater work function impairment. Following a previous study, we categorized WFun scores into four groups: no work function impairment (7–13 points), low work function impairment (14–20 points), moderate work function impairment (21–27 points), and severe work function impairment (28–35 points).^21^ WFun scores strongly reflect musculoskeletal pain intensity levels.^22^

### 2.4 Coping behaviors for low back pain

Subjects were then asked if they were engaging in any coping behaviors for the health problem that was affecting their work the most (i.e., back pain) and asked to select all coping behaviors for the symptom. Coping behaviors were described as follows: (1) I am receiving or have received medical attention/treatment from a physician, (2) I take or have taken over‐the‐counter drugs, and (3) I take care of myself by stretching, exercises, or massage on a daily basis. Possible responses were “yes” and “no.”

### 2.5 Covariates

Information about age, sex, type of occupation, employment status, and overtime hours was collected from the questionnaire. Age as of March 31, 2017, was measured as a continuous variable. We divided respondents into 10‐year age categories. Sex was categorized as male or female. The type of occupation was defined as clerical and administrative support, sales, research and development, production line, and others. The amount of overtime work was assessed using the following question: “What was your average number of overtime work hours per month during the most recent 6 months? Please choose the most appropriate option (include holiday work hours; do not include commuting time).” There were 12 response options: 0, <10, 10–19, 20–29, 30–39, 40–49, 50–59, 60–69, 70–79, 80–89, 90–99, and ≥100 h. Based on the statistical distribution, average overtime hours in the last 6 months were categorized into six groups: 0, <10, 10–19, 20–29, 30–39, and ≥40 h.

### 2.6 Statistical analysis

Logistic regression analysis was performed to identify the association between work function impairment level and three coping behaviors. We performed logistic regression analysis on responses to the three coping behavior questions (seeking medical attention and treatment by a physician, taking over‐the‐counter drugs, and self‐care by stretching, exercises, and massage) as the outcome variable and WFun score as the explanatory variable, with 1 assigned for each behavior performed and 0 assigned for each behavior not performed. Odds ratios and 95% confidence intervals (95% CI) were calculated for low, moderate, and severe work function impairment using no work function impairment as a reference. We performed a crude analysis with no adjustment and an analysis adjusted for sex, age, type of occupation (categorical variable), and monthly average overtime hours in the last 6 months (categorical variable). We thought there might be an effect of interaction between coping behaviors, so we conducted a sensitivity analysis. That was conducted, in each of the three logistic regression analyses, by adding two variables regarding coping behavior other than the outcome variable were used as explanatory variables to each adjusted analysis.

All statistical analyses were performed using Stata/IC version 16.0 (StataCorp LLC). We used two‐sided statistical tests, and a probability value of *p* < .05 was regarded as statistically significant.

### 2.7 Ethics

The study was explained to business owners and workers via email and intranet. It was explained in advance that if they did not agree with the study purpose, they could choose to not respond and would not be disadvantaged for this. This study was approved by the ethics committee of the University of Occupational and Environmental Health, Japan (H26‐026) and was conducted in full accordance with the World Medical Association Declaration of Helsinki.

## 3 RESULTS

**FIGURE 1:**
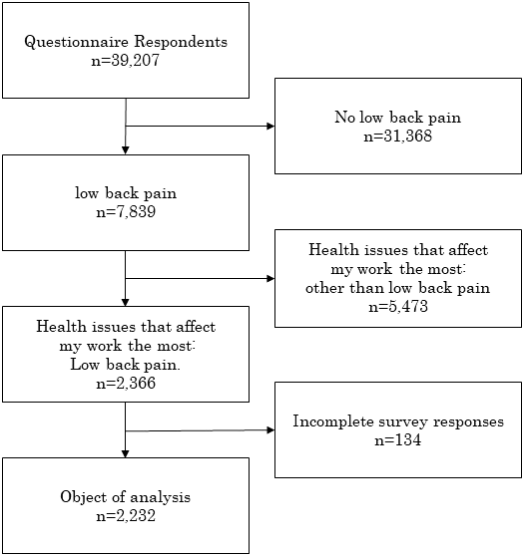
Flow diagram of research subject selection

The questionnaire was distributed to 53 780 people (53 317 in the manufacturing companies and 463 in the medical and the welfare service company). A total of 39 207 workers (38 938 in the manufacturing industry and 269 in the medical and the welfare service company) aged 20 years or above agreed to the research objectives and responded to the questionnaire. The total response rate was 72.9% (73.0% in the manufacturing industry and 58.1% in the welfare service company). Of individuals who responded to the questionnaire, 7839 reported back pain as one of several health problems. In those who had back pain, 2366 chose back pain among other comorbid health problems as the health problem that most affected their work. After excluding 134 respondents who did not complete the questionnaire, 2232 people were included in the analysis (Figure 1).

Table 1 shows the subject characteristics. Most subjects were men. One‐third of respondents were in their 40s, and the average age was 44.3 years. As the survey was mainly conducted in the manufacturing industry, most respondents worked on production lines. More than 80% of respondents worked less than 40 h of overtime per month on average, and more than half of respondents scored 7–13 on the WFun scale, suggesting that they had no problems with occupational dysfunction. The most frequent coping behavior was self‐care. 54.9% of the subjects responded that they were doing the behavior. In addition, 30.8% of the subjects had visited a medical institution and 19.5% of the subjects had taken over‐the‐counter medication as a coping behavior in the past (Table 1).

**TABLE 1:** Demographic characteristics of the study population

	N	%
WFun score		
7–13	1,281	57.4
14–20	611	27.4
21–27	252	11.3
28–35	88	3.9
Sex		
Men	2,006	90.0
Women	226	10.0
Age, years		
20–29	262	11.7
30–39	459	20.6
40–49	741	33.2
50–59	615	27.6
60≤	155	6.9
Occupation		
Clerical and administrative support	243	10.9
Sales	334	15.0
Research and development	333	14.9
Production line	790	35.4
Others	138	6.2
Unkown	394	17.7
Average overtime hours in the last 6 months		
0	118	5.3
1–9	482	21.6
10–19	407	18.2
20–29	459	20.6
30–39	347	15.6
40‐	419	18.8
Coping behaviors for low back pain		
Seeking medical attention and treatment by a physician	688	30.8
Taking over‐the‐counter drugs	436	19.5
Taking care of myself by stretching, exercises, and massage	1225	54.9

Table 2 shows the relationship between work function impairment level and seeking medical attention and treatment by a physician. About 30% of those in the no, low, or moderate work functional impairment group visited a medical institution. On the other hand, more than half of those in the severe group visited a medical institution. Subjects experiencing severe work function impairment were more likely to seek medical attention and physician treatment than those with no work function impairment. The multivariate‐adjusted analysis showed that the adjusted odds ratio for seeking medical attention and physician treatment was 2.84 (95% CI: 1.82–4.45, *p* < .001) for severe work function impairment, compared with no work function impairment. The results of the sensitivity analysis added explanatory variables about other coping behaviors, that were whether objects used over‐the‐counter drugs, and were taking self‐care, also showed similar results, with odds ratios of 1.21 (*p* = 1.51, 95% CI: 0.98–1.51) for low, 1.06 (*p* = 1.45, 95% CI: 0.76–1.45) for moderate, and 3.04 (*p* < .001, 95% CI: 1.92–4.82) for severe, with no problem as the reference.

**TABLE 2:** The relationship between the degree of work functioning impairment and the behavior of seeking medical attention and treatment by a physician

	Total	Workers who answered yes		Crude analysis			Adjusted analysis		
	N	N	%	OR	95% CI	*p* value	OR	95% CI	*p* value
No work functioing impairment (WFun score 7–13)	1281	376	29.4	reference			reference		
Low work functioning impairment (WFun score 14–20)	611	193	31.6	1.11	0.90–1.37	.32	1.18	0.95–1.46	.13
Moderate work functioning impairment (WFun score 21–27)	252	74	29.4	1.00	0.74–1.35	1.00	1.09	0.80–1.48	.59
Severe work functioning impairment (WFun score 28–35)	88	45	51.1	2.52	1.63–3.89	<.001	2.84	1.82–4.45	<.001

Adjusted analysis: adjusted for sex, age, occupation(category), and average overtime hours in the last 6 months(category).

Abbreviations: CI, Confidential interval; OR, odds ratio; WFun, work functional questionnaire.

Table 3 shows the relationship between work function impairment level and taking over‐the‐counter drugs. It was confirmed that the utilization rate of over‐the‐counter medication increased as the degree of work functional impairment increased. The utilization rate of over‐the‐counter medication was 17.8% for no problem, 20.6% for low, 23.0% for moderate, and 27.3% for severe. The results of the adjusted analysis showed that the adjusted odds ratio for use of over‐the‐counter drugs was 1.65 (95% CI: 1.00–2.73, *p* = .05) for severe work function impairment, compared with no work function impairment. Although there was no statistically significant difference in the adjusted odds ratios, we observed a trend for the use of over‐the‐counter drugs with higher levels of work function impairment. The results of the sensitivity analysis also showed the similar trend, with odds ratios of 1.22 (*p* = .13, 95% CI: 0.95–1.56) for low, 1.34 (*p* = .10, 95% CI: 0.96–1.88) for moderate, and 1.89 (*p* = .01, 95% CI: 1.14–3.15) for severe, with no problem as the reference.

**TABLE 3:** The relationship between the degree of work functioning impairment and the behavior of taking over‐the‐counter drugs

	Total	Workers who answered yes		Crude analysis			Adjusted analysis		
	N	N	%	OR	95% CI	*p* value	OR	95% CI	*p* value
No work functioing impairment (WFun score 7–13)	1281	228	17.8	reference			reference		
Low work functioning impairment (WFun score 14–20)	611	126	20.6	1.20	0.94–1.53	.14	1.19	0.92–1.52	.18
Moderate work functioning impairment (WFun score 21–27)	252	58	23.0	1.38	1.00–1.91	.05	1.35	0.96–1.89	.09
Severe work functioning impairment (WFun score 28–35)	88	24	27.3	1.73	1.06–2.83	<.05	1.65	1.00–2.73	.05

Adjusted analysis: adjusted for sex, age, occupation(category), and average overtime hours in the last 6 months(category).

Abbreviations: CI, Confidential interval; OR, odds ratio; WFun, work functional questionnaire.

Table 4 shows the relationship between work function impairment level and self‐care by stretching, exercises, and massage. The rate of self‐care was approximately 50%–55% in the four groups by work functional impairment. Statistically significant results could not be obtained in the adjusted analysis. The results did not indicate that individuals with any level of work function impairment engaged in self‐care more than those with no work function impairment. The results of the sensitivity analysis also showed no difference in results, with odds ratios of 1.09 (*p* = .39, 95% CI: 0.89–1.34) for low, 0.80 (*p* = .12, 95% CI: 0.60–1.06) for moderate, and 1.08 (*p* = .73, 95% CI: 0.69–1.70) for severe, with no problem as the reference.

**TABLE 4:** The relationship between the degree of work functioning impairment and the behavior of taking care of myself by stretching, exercises, and massage

	Total	Workers who answered yes		Crude analysis			Adjusted analysis		
	N	N	%	OR	95% CI	*p* value	OR	95% CI	*p* value
No work functioing impairment (WFun score 7–13)	1281	716	55.9	reference			reference		
Low work functioning impairment (WFun score 14–20)	611	344	56.3	1.02	0.84–1.23	.87	1.05	0.86–1.28	.64
Moderate work functioning impairment (WFun score 21–27)	252	121	48.0	0.73	0.56–0.96	<.05	0.80	0.61–1.06	.12
Severe work functioning impairment (WFun score 28–35)	88	44	50.0	0.79	0.51–1.22	.28	0.87	0.56–1.35	.53

Adjusted analysis: adjusted for sex, age, occupation(category), and average overtime hours in the last 6 months(category).

Abbreviations: CI, Confidential interval; OR, odds ratio; WFun, work functional questionnaire.

## 4 DISCUSSION

In this study, we identified the behavioral patterns of workers with low back pain. More than 50% of those with severe work functional impairment had visited a medical institution. There was a relationship between having severe work dysfunction and seeking medical care. Over‐the‐counter medication was correlated with the level of work functional impairment. This tendency was observed even when the effects of possible confounding factors such as gender and age were removed. No clear relationship was found between occupational dysfunction and self‐care.

These behavioral traits for seeking medical care and taking over‐the‐counter drugs are consistent with those found in previous studies. Studies of patients with chronic low back pain show an association between the number of medical visits in a 6‐month period and greater pain intensity, and an association between the rate of over‐the‐counter drug use in the previous 6 months and greater pain intensity.^23^, ^24^ Since this study was a cross‐sectional study, it is not possible to point out a causal relationship, but considering the relationship between the presence of low back pain and attempts to seek medical attention or take over‐the‐counter medication, a high level of labor dysfunction may promote seeking medical attention or using over‐the‐counter medication.

In this study, there was no apparent relationship between work dysfunction and self‐care. About half of the subjects were engaged in self‐care. According to previous studies, the factor that encourages self‐care is not pain, but self‐elevation.^25^ Considering that WFun scale is a strong reflection of pain indices, the results of this study do not contradict previous studies.^22^ In other words, self‐care may not necessarily be encouraged if the degree of work functional disability is severe. It is necessary to further examine the factors that promote self‐care for workers with low back pain.

The important point identified in this study is that nearly half of them do not engage in self‐care, and self‐care does not seem to be facilitated by the strong work performance decline of low back pain. Self‐care is recommended for almost all patients with low back pain, unless there are contraindications.^26^ This is an important issue for companies because disability caused by low back pain results in substantial presenteeism costs. Workplace self‐care programs for low back pain have been conducted as health promotion activities. A combined program of yoga and strength training in the workplace did not reduce sickness absence in the population more than evidence‐based advice alone, but the workers who did it at least twice a week showed a significant reduction in sickness absence.^27^ Self‐management programs for low back pain are effective in improving functional disability, and supervised exercise therapy is thought to be most effective, but constant monitoring is difficult, so providing strategies to encourage adherence is recommended.^28^, ^29^ These findings indicate the importance of promoting self‐care for workers who experience occupational functional disability caused by low back pain.

There are several study limitations. The first is that because this was a cross‐sectional study, it was not possible to test for causality. It is unclear whether subjects developed work disability from lack of exercise, or whether increased work function impairment caused by back pain led to the avoidance of self‐care. We performed the sensitivity analysis to consider the effect of unmeasured confounders, but we did not find any results that would affect our discussion. Second, the internal validity of the research method was compromised. This is because the purpose of this study was to study the coping behaviors of workers with low back pain, but in the selection of subjects, workers who answered that low back pain was the symptom that most affected their work were included. Those whose work performance is affected by low back pain are more selective than those who simply have low back pain, so it is difficult to find a significant difference. Nevertheless, our study found them, suggesting that there is the strong relationships between the work functional impairment and coping behaviors. Third, the cause of back pain was unknown. The natural course of low back pain differs depending on its cause and may therefore require a range of different treatment methods. Additionally, we should have had exclusion criteria for low back pain (e.g., pregnant women and cancer patients). Fourthly, there are limitations to the content validity of the data in this study. Because the subjects were asked to complete a self‐administered questionnaire, we were unable to verify the validity of the data regard to coping behaviors. Further research using objective data, such as data on medical insurance applications, is needed to determine whether or not workers are seeing medical institutions.

The greatest strength of this study is that it is the first to report on the relationship between work function impairment and coping behaviors. By investigating three coping behaviors with different characteristics, we were able to clarify the behavioral characteristics of workers with impaired functioning owing to low back pain. In addition, we tried to eliminate as many confounding factors as possible by adjusting for factors such as age, sex, and occupation. Finally, this was a large‐scale survey across 14 companies and had a high response rate.

## 5 CONCLUSIONS

We were able to clarify the relationship between work function impairment level and three coping behaviors in workers with low back pain. The fact that about half of workers with low back pain do not engage in self‐care is a serious issue for companies experiencing presenteeism costs, and it is necessary to provide workers with appropriate disability support. Further research is needed to evaluate whether interventions that promote self‐coping for workers with work function impairment can reduce presenteeism costs.

## DISCLOSURE

*Approval of the research protocol*: This study was approved by the ethics committee of the University of Occupational and Environmental Health, Kitakyushu, Japan, and was conducted in full accordance with the World Medical Association Declaration of Helsinki. *Informed consent*: We explained the study protocol to subjects and obtained opt‐out consent. *Registry and the registration no. of the study/trial*: N/A. *Animal studies*: N/A. *Conflict of interest*: The authors declare that they have no conflict of interest.

## AUTHOR CONTRIBUTIONS

TN and YF conceived the research idea; MN, SK, and TN collected the data; KS and TN analyzed the data; and KS and KM led the writing of the manuscript. All authors participated in critically reviewing the study.

## Data Availability

The data used in this study is not publicly available and please contact us for more information.
